# Multicellularity in green algae: upsizing in a walled complex

**DOI:** 10.3389/fpls.2014.00649

**Published:** 2014-11-18

**Authors:** David S. Domozych, Catherine E. Domozych

**Affiliations:** Skidmore Microscopy Imaging Center, Department of Biology, Skidmore College, Saratoga SpringsNY, USA

**Keywords:** cell wall, extracellular matrix, multicellularity, pectin, glycoprotein, cytokinesis

## Abstract

Modern green algae constitute a large and diverse taxonomic assemblage that encompasses many multicellular phenotypes including colonial, filamentous, and parenchymatous forms. In all multicellular green algae, each cell is surrounded by an extracellular matrix (ECM), most often in the form of a cell wall. Volvocalean taxa like *Volvox* have an elaborate, gel-like, hydroxyproline rich glycoprotein covering that contains the cells of the colony. In “ulvophytes,” uronic acid-rich and sulfated polysaccharides are the likely adhesion agents that maintain the multicellular habit. Charophytes also produce polysaccharide-rich cell walls and in late divergent taxa, pectin plays a critical role in cell adhesion in the multicellular complex. Cell walls are products of coordinated interaction of membrane trafficking, cytoskeletal dynamics and the cell’s signal transduction machinery responding both to precise internal clocks and external environmental cues. Most often, these activities must be synchronized with the secretion, deposition and remodeling of the polymers of the ECM. Rapid advances in molecular genetics, cell biology and cell wall biochemistry of green algae will soon provide new insights into the evolution and subcellular processes leading to multicellularity.

## INTRODUCTION

A multicellular organism consists of an organized aggregation of cells that are products of geometrically patterned cell divisions which maintain physical communication networks with each other. The evolution of the multicellular form or phenotype has occurred in multiple and diverse assemblages of eukaryotes distributed over the kingdoms of life. It is widely accepted that multicellularity evolved six times in modern photosynthetic eukaryotes including twice in the red algae (Rhodophyta), twice in the photosynthetic stramenopiles (e.g., brown algae or Phaeophyta) and twice in the green algae (Niklas, 2014). The multicellular phenotypes exhibited in modern day green algae are quite diverse and are exemplified by colonies, unbranched and branched filaments, and parenchymatous thalli (Graham et al., 2009). The evolution of these multicellular forms in green algae has been of profound importance to the natural history of the planet. In the charophyte lineage (e.g., Streptophyta; Leliaert et al., 2012) of green algae, one ancient multicellular form emerged onto land approximately 450–500 million years ago and ultimately yielded land plants, i.e., a transformative event that changed the biogeochemistry of the planet. One of the key cellular features of multicellular green algae as well as all other multicellular photosynthetic eukaryotes is the presence of an extracellular matrix (ECM) that is positioned on the external face of the plasma membrane of each cell. Most often, the ECM is expressed in the form of a highly complex composite of fibrillar and matrix polymers called a *cell wall*. Each cell of multicellular green algae and their descendants, the land plants, produces a cell wall that must expand and chemically modulate in coordination with neighboring cells. The production of the cell wall during cell division requires significant contributions of the cell’s membrane trafficking and cytoskeletal networks that are carefully regulated by complex gene expression programs and signal transduction cascades reacting to external stresses (e.g., light, temperature, contact with a pathogen). The cell division mechanism must also create and maintain intercellular symplastic connections through the cell walls of adjacent cells throughout the life cycle so as to establish an effective intercellular communication network necessary for multicellular life.

Recent investigations based on molecular, biochemical, developmental and cell biology-based studies have provided significant insight into the evolution of multicellularity in green algae and the subsequent origin of land plants. Many outstanding reviews are available that summarize these findings (Niklas, 2004, 2014; Bennici, 2008; Leliaert et al., 2012; Pires and Dolan, 2012; Niklas and Newman, 2013; Niklas et al., 2013; Zhong et al., 2013). This paper focuses on the role of the cell wall and its inclusive polymers in the development and evolution of multicellular green algae. Significant differences exist in polymer composition of the cell walls of the major green algal lineages expressing multicellularity (e.g., volvocine forms from the chlorophyte line vs. charophytes). However, recent studies have also demonstrated that remarkable similarity exists in cell wall composition of late divergent charophyte green algae with land plants (Domozych et al., 2007, 2012; Eder and Lütz-Meindl, 2008, 2010; Popper and Tuohy, 2010; Sørensen et al., 2010, 2011). It is apparent that the evolution of the cell wall was critical in the evolution of the multicellular phenotype. Furthermore, pre-adaptation of cell wall composition and architecture in ancient multicellular charophytes was most likely very important to the colonization and exploitation of terrestrial habitats.

## VOLOCINE MULTICELLULARITY AND THE GLYCOPROTEIN ECM

One of the earliest studied examples of the unicellular-to-multicellular transition in green algae is the chlorophyte group, Volvocales (Miller, 2010; Leliaert et al., 2012). This is an assemblage of organisms that exhibit flagella-generated motility during a major part of their life cycles. The Volvocales include both unicellular and multicellular taxa. In the latter, multicellularity is exhibited in colonies that have permanent or transient cytoplasmic connections and coordinated communication networks between cells.

**FIGURE 1:**
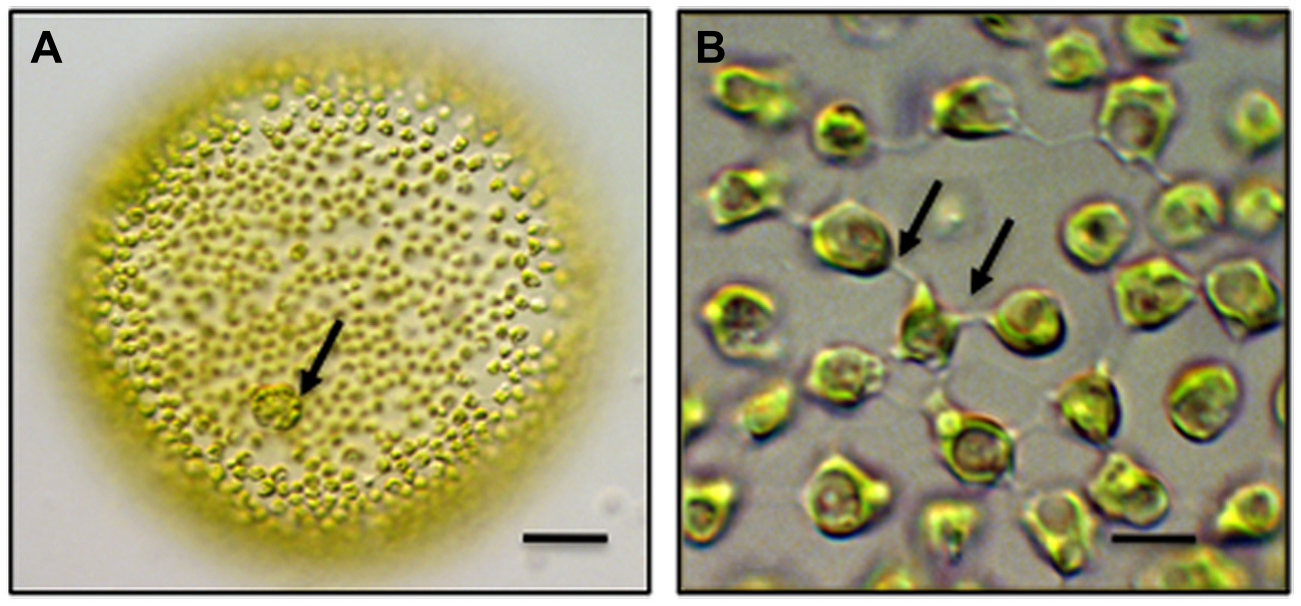
**Multicellular *Volvox globator*. (A)** A colony consists of over 2,000 cells. Biflagellated somatic cells line the exterior of the colony and gonidia (arrow) are embedded within the extracellular matrix (ECM). bar = 100 μm. **(B)** Intercellular bridges (arrows) connect the somatic cells of colony and penetrate the surrounding ECM. bar = 6 μm. Differential interference contrast (DIC) light microscopy (LM) images.

*Chlamydomonas* is the most well-known and – studied unicellular volvocine genus and contains approximately 600 species. *Chlamydomonas* is a spherical unicell with two anterior flagella (Hallman, 2006). Cell division in *Chlamydomonas* yields four daughter protoplasts, each of which produces a cell wall before being released through the ruptured parental cell wall. Multicellular taxa of the Volvocales include *Gonium*, *Pandorina* and most well-known, *Volvox*. A *Volvox* colony consists of two cell types. First, approximately 2,000–4,000 bi-flagellated somatic cells, each similar in morphology to *Chlamydomonas*, form a single layer that lines the outer surface of the colony (**Figure 1**). These cells beat their flagella in synchrony to produce a coordinated rolling motility of the colony in liquid medium. The somatic cells are terminally differentiated in that they do not undergo cell division. Interior to this layer are up to 16 reproductive cells or gonidia. Each gonidium will undergo 11–12 synchronized cell divisions to form a daughter colony positioned in the parent colony (Hoops et al., 2006). Gonidial cytokinesis is phycoplast-mediated (Green and Kirk, 1981; Green et al., 1981) but is incomplete resulting in multiple physical connections in the form of narrow cytoplasmic bridges or strands between cells. In *Volvox carteri*, each bridge is approximately 200 nm in diameter and separated from adjacent bridges by 500 nm. The bridges are positioned in a concentric ring on the inner surface of the colony and are believed to hold the entire embryo together. Each of these strands is lined by plasma membrane and contains cytoplasm often with transient cytoskeletal components that bridge adjacent cells (Hoops et al., 2006). A daughter colony contains approximately 100,000 bridges with each cell connected to an adjacent cell by about 25 bridges. The two flagella produced in each of the cells of the daughter colony are positioned inward. Daughter colony release from the parent colony entails an inversion process so that the flagella are ultimately positioned on the outside of the colony. The cytoplasmic bridges between the cells are retained during inversion and are believed to serve as braces for holding cells together in the colony during inversion. In about one-half of all colonial volvocine species, these cytoplasmic connections remain intact, become broader and number 4–6 between cells. The cytoplasmc bridges are thought to be conduits for signal transduction in coordinating cell division in the developing daughter colony. The cells of the colony are embedded in a distinct ECM often consisting of a cell wall and a gelatinous matrix.

The “cell wall” of volvocine algae is very different from those found in the charophytes and land plants, most notably in that it is devoid of large and complex networks of polysaccharides. Rather, the wall consists of a complex network of 25–30 hydroxyproline-rich glycoproteins (i.e., HRGPs), some similar to extensin, that form a weak non-covalent wall lattice (Keskiaho et al., 2007; Lamport et al., 2011). These HRGPs self assemble into dense fibrous meshworks that are stablilized by cross-linking (Ferris et al., 2005). High resolution TEM imaging of cryo-processed *Chlamydomonas* cell walls reveals a crystalline outer layer that can be extracted by chaotropic agents and a thick, inner insoluble layer (Voigt et al., 2007). In *Volvox*, the wall/ECM is composed of at least four distinct geographic regions with some of the HRGPs elaborating into a gel-like sheath (Kirk et al., 1986; Ertl et al., 1989, 1992). One major family of HRGPs of the cell walls of volvocine taxa is the “pherophorins” (Hallman, 2006). The proteins of this family exhibit a hydroxyproline-rich rod-like domain with surrounding globular domains at its two termini that is similar to the Solanaceae lectin class of extensins. It is thought that this lectin-like carbohydrate-binding ability provides cross-linking capability in the wall/ECM (i.e., adhesion).

The common ancestor of unicellular and multicellular volvocine algae diverged relatively recently, i.e., 50–200 million years (Herron et al., 2009). Likewise, comparative molecular analyses have shown that *Chlamydomonas* and *Volvox* genomes are remarkably similar (Prochnik et al., 2010). These two features have enhanced the identification of key characteristics that separate extant unicellular and multicellular taxa and those that may have been critical to the evolution of the multicellular form. The *Volvox* genome is approximately 17% larger than that of *Chlamydomonas.* This is due in part to *Volvox*’s greater transposon/repetitive DNA content, but more significantly, to *Volvox*’s increased numbers of proteins, primarily those associated with an expanded and highly compartmentalized ECM/cell wall (Prochnik et al., 2010). In fact, it is estimated that each *Volvox* cell produces an ECM that is 10,000 times larger than the ECM/wall of a *Chlamydomonas* cell (Abedin and King, 2010; Blaby et al., 2014). This strongly suggests that major elaborations of the ECM/cell wall were critical in the evolution of the multicellular habit in volvocine algae. ECM/wall components form the structural framework that provides the resistive force that counterbalances turgor pressure which would otherwise make formation/maintenance of the cytoplasmic bridges impossible. Likewise, the elaboration of the lectin-like pherophorins and their carbohydrate-binding capability in the ECM may very well be the glue that keeps the multicell aggregation of cells in the colony together. Interestingly, the HRGPs of multicellular volvocine taxa are not highly cross-linked especially when compared to extensins in land plants. Therefore, this ECM framework cannot support large and complex networks of structural polysaccharides. This is believed to limit the size of expansion of volvocine taxa (Lamport et al., 2011). It is also interesting to note that some HRGPs of both multicellular and unicellular volvocine taxa have evolved into key macromolecules used for sexual signaling (e.g., sex inducers, sexual agglutinins; Ender et al., 1999; Ferris et al., 2005; Lee et al., 2007). The number of these is also much greater in *Volvox* than in unicellular volvocine taxa. This has led to the supposition that during the evolution of the multicellular form, ECM/cell wall proteins also diversified and most likely were recruited into developmental processes (e.g., sexual reproduction), thus representing a source of adaptive plasticity that is specific to the volvocine algae (Prochnik et al., 2010).

## MULTICELLULARITY IN OTHER CHLOROPHYTES: WALLED

The Volvocales represent just one of the many taxonomic groups in the diverse chlorophyte line of evolution in the green algae. Unlike the volvocine group though, there is a paucity of information on, and comparative studies of, the chemical nature of the cell wall of these algae (see Domozych et al., 2012) and few genomes have been thoroughly analyzed. Multicellular thalli are found in the chlorophytes including branched and unbranched filaments as well as filamentous/parechymatous sheet-like thalli of the *Ulva*-Ulotrichales-Trentopohliales group (Cocquyt et al., 2010; Leliaert et al., 2012). Additionally, there are some taxa that produce large thalli but represent multinucleate cells that are products of uncoupled cytokinesis and mitosis, i.e., the siphonocladous ulvophytes. These organisms have external cell walls but no cross walls. However, cytoplasmic domains containing a nucleus are individualized. Within the diverse group of multicellular chlorophytes, many cell wall types exist. In the freshwater filamentous taxon, *Oedogonium*, it has been shown that cellulose, pectins, including homogalacturonans (HGs) and rhamnogalcturonan I (RGI), and HRGPs like extensin and arabinogalactan proteins (AGPs) are present in the cell wall (Estevez et al., 2008). The role of these polymers in cell–cell adhesion may be similar to the charophytes (see below) but further studies are clearly needed to resolve this. In marine green seaweeds, there is much diversity in cell wall chemistry. For example in *Codium*, sulfated glucuronoxylomannans, glucuronoxylorhamnogalactans and) sulfated xyloarabinogalactans are major cell wall components (Estevez et al., 2009; Fernandez et al., 2010). In the related *Bryopsis*, sulfated galactans and rhamnans are also major wall constituents (Ciancia et al., 2012). In *Ulva*, a main constituent of the cell wall is ulvan whose backbone structure includes sulfated rhamnose residues linked to uronic acids, resulting in a repeated disaccharide unit β-D-glucuronosyl-(1,4)-α-L-rhamnose 3-sulfate, called aldobiouronic acid (Lahaye and Robic, 2010). This polysaccharide is found in spaces between adjacent cells (Bobin-Dubigeon et al., 1997), i.e., putative cell–cell adhesion zones. In the related genus, *Monostroma*, sulfated rhamnans are also found in the cell walls (Mao et al., 2008). The role of the cell wall and inclusive components in maintaining the cell–cell adhesive network in multicellular chlorophytes awaits further study. However, the abundance of uronic acid-rich and/or sulfated polysaccharides in the cell wall matrix may indicate that they are key components in maintenance of wall microarchitecture and forming the framework of the multicellular thallus.

## CHAROPHYTE MULTICELLULARITY AND THE JOURNEY TO LAND PLANTS

The charophyte or streptophyte (Streptophyta) clade is the lineage of green algae that is ancestral and most closely related to land plants (Leliaert et al., 2012). Modern charophytes exhibit a wide range of morphological forms including unicell (*Mesostigma*), sarcinoid packet (aggregations of 4–8 cells but with no intercellular connections; *Chlorokybus*) and diverse multicellular thalli that includes unbranched filaments (*Klebsormidium*, *Spirogyra*, *Mougeotia*, filamentous desmids), branched filaments (*Coleochaete nitellarum*), filamentous aggregates that form 3-dimensional thalli (*Chara*) and pseudoparenchymatous forms (*Coleochaete orbicularis*). Many multicellular charophytes also exhibit notable structural and developmental characteristics that are also found in land plants. For example, in many multicellular charophytes, intercellular connections in the form of plasmodesmata penetrate cell walls and join adjacent cells (Cook and Graham, 1999). The plasmodesmata are primarily formed during cell division, specifically due to interruptions to the phragmoplast-cell plate mechanism in a process similar to that of land plants. Some charophytes exhibit developmental processes that also lead to dorsiventral symmetry, a morphogenetic process that is commonly found in land plants. Some taxa of the late divergent clades, i.e., the Coleochaetales and Charales, also produce multicellular gametangia such as oogonia and antheridia that are quite similar in construction to gametangia of land plants. Finally, recent molecular studies of charophytes have identified the biosynthetic pathways for the synthesis and perception of several hormones that were previously thought to be found only in land plants (e.g., strigolactones, ethylene; De Smet et al., 2010; Delaux et al., 2012; Hori et al., 2014). All of these characteristics demonstrate that charophyte multicellularity is quite complex and that several ancient taxa evolved pre-adaptive mechanisms for the exploration, invasion and conquest of land 450–500 million years ago.

All multicellular charophytes possess cell walls that are composed of an assortment of neutral and acidic polysaccharides along with various glycoproteins, i.e., a condition very different than the wall composition of volvocine taxa. Taxa of the late divergent charophyte clades (e.g., Zygnematales, Coleochaetales, Charales) have remarkably similar polymer composition to the cell walls of many land plants (Sørensen et al., 2010, 2011) and it is currently presumed that these polymers are most likely incorporated into the *basic* microarchitectural design of the wall in a similar fashion to the cell walls of land plants. The current microarchitectural model of the cell wall is one of a fibrous composite with cellulose microfibrils forming the load-bearing component of the wall (Cosgrove and Jarvis, 2012). The microfibrils are tethered by various hemicelluloses and embedded in a matrix of pectins and proteins including extensin and AGPs (Burton et al., 2010). Various enzymes (e.g., pectin methylesterase or PME, Xyloglucan Endotransglycosylase or XET, wall-associated kinases, WAKS), non-enzymatic proteins (e.g., expansin), ions (e.g., Ca^2+^) and water also contribute to the structure and development of the wall (Cosgrove, 2005; Eklof and Brumer, 2010; Jolie et al., 2010; Frankova’ and Fry, 2013; Fry, 2011; Liu et al., 2013).

It is quite apparent that the outer layers of the wall of any cell in a multicellular system represent the physical contact points with the cell walls of adjacent cells and accommodate cell–cell adhesion. In the thallus of multicellular charophytes and land plants, cells are always found attached to adjacent cells via components of their cells walls, i.e., from the time they are born as daughter cells to their terminal differentiated forms (Jarvis et al., 2003). Cell–cell attachment is under constant pressure by turgor. Turgor pressure is the force necessary for regulating expansion and providing mechanical rigidity. However, it also creates formidable stress that can shear adjacent cell walls apart and separate cells. This is combated by cells reinforcing specific zones of their cell walls that are located at points of maximal stress and include the cell corners (tricellular junctions; *sensu* Jarvis et al., 2003) and the middle lamella found between adjacent cell walls (Caffall and Mohnen, 2009). In the cell walls at these loci, specific polymers are incorporated to promote the adhesion efficacy. This adhesion mechanism is also a critical part of cell expansion of young daughter cells as highly coordinated wall polymer secretion and modifications must occur between neighboring cells in order to ensure that their adherent walls remain fused.

The class of wall polysaccharides that is commonly found in the middle lamella and cell junctions and one that has been directly linked to binding adjacent cells in multicellular tissues is pectin. Pectin constitutes a diverse group of galacturonic acid (GalA)- containing polysaccharides that make up a significant portion of the matrix of the cell wall (Mohnen, 2008; Caffall and Mohnen, 2009). Pectin consists of three major subclasses: HG, substituted HGs (e.g., xylogalacturonans, rhamnogalacturonan II), and rhamnogalacturonan I (RG-I; Willats et al., 2001; Mohnen, 2008). HG is the most common of pectic polysaccharides constituting greater than 60% of the pectin of primary plant walls (Caffall and Mohnen, 2009). HG is composed of a linear chain of α-1,4-linked GalA residues that are often methylesterified on C6 or acetylated on C2 and/or C3. HG is the main pectin found in key adhesion zones of multicellular plants and its post-secretion modulation dynamics provide insight into its importance in adhesion (Domozych et al., 2014). HG is thought to be synthesized in a highly methylesterified form in the medial locus of Golgi apparatus and transported to the cell surface by the secretory vesicle network (Held et al., 2011; Driouich et al., 2012). When secreted into the wall, the HG is de-esterified by enzymes such as pectin methyl esterase or PME. This action exposes a negatively charged carboxyl group at C-6 of the GalA residue. This subsequently allows for a complexing or crosslinking of the GalAs of adjacent pectin chains with cations like Ca^2+^ to form stable gels with HG chains in a tightly packed conformation. This occurs only if 10 or more consecutive un-methylesterified GalA residues are coordinated in each chain (i.e., available for the cross-linking). It is very likely that these Ca^2+^-HG complexes create a stable, 3-dimensional adhesive network between adjacent cells in the multicellular system. Likewise, while this cross-linked network may only include HGs, it appears more likely that this adhesive network includes pectic chains covalently linked to other insoluble polysaccharides in the cell walls of the adjacent cells. For example, HG may be just one part of a larger or super pectin macromolecule that also contains RG-I. RG-I is made of repeating subunits of -> α -D-GalA-1,2- α -l-Rhamnose 1,4- that may be substituted with unbranched or branched arabinan, galactan, or arabinogalactan side chains. The RG-I is covalently linked to the HG part of the super pectin macromolecule. Recently, exciting new data (Tan et al., 2013) show that in *Arabidopsis*, the RG-I component of the super pectin complex is attached to AGP and arabinoxylan in the wall. Earlier work showed that side chains of the RG-I bind to cellulose microfibrils (Zykwinska et al., 2005, 2007). This evidence clearly shows direct links between the diverse set of wall polysaccharides and suggests that branched RG-I contributes to cell wall-based cellular adhesion in multicell thalli (Mendu et al., 2011; Agoda-Tandjawa et al., 2012). At this time, a working model of the microarchitecture of adhesion zones of cell walls in the multicellular system would still be based on Ca^2+^-HG cross-linking but would also include multiple connections with other wall polymers via RG-I to form a strong network that can resist the forces of expansion fueled by turgor. Future work on identifying the inclusive interpolymeric associations of the middle lamella and cell junctions will provide critical insight into the maintenance of multicellular condition in charophytes and land plants.

**FIGURE 2:**
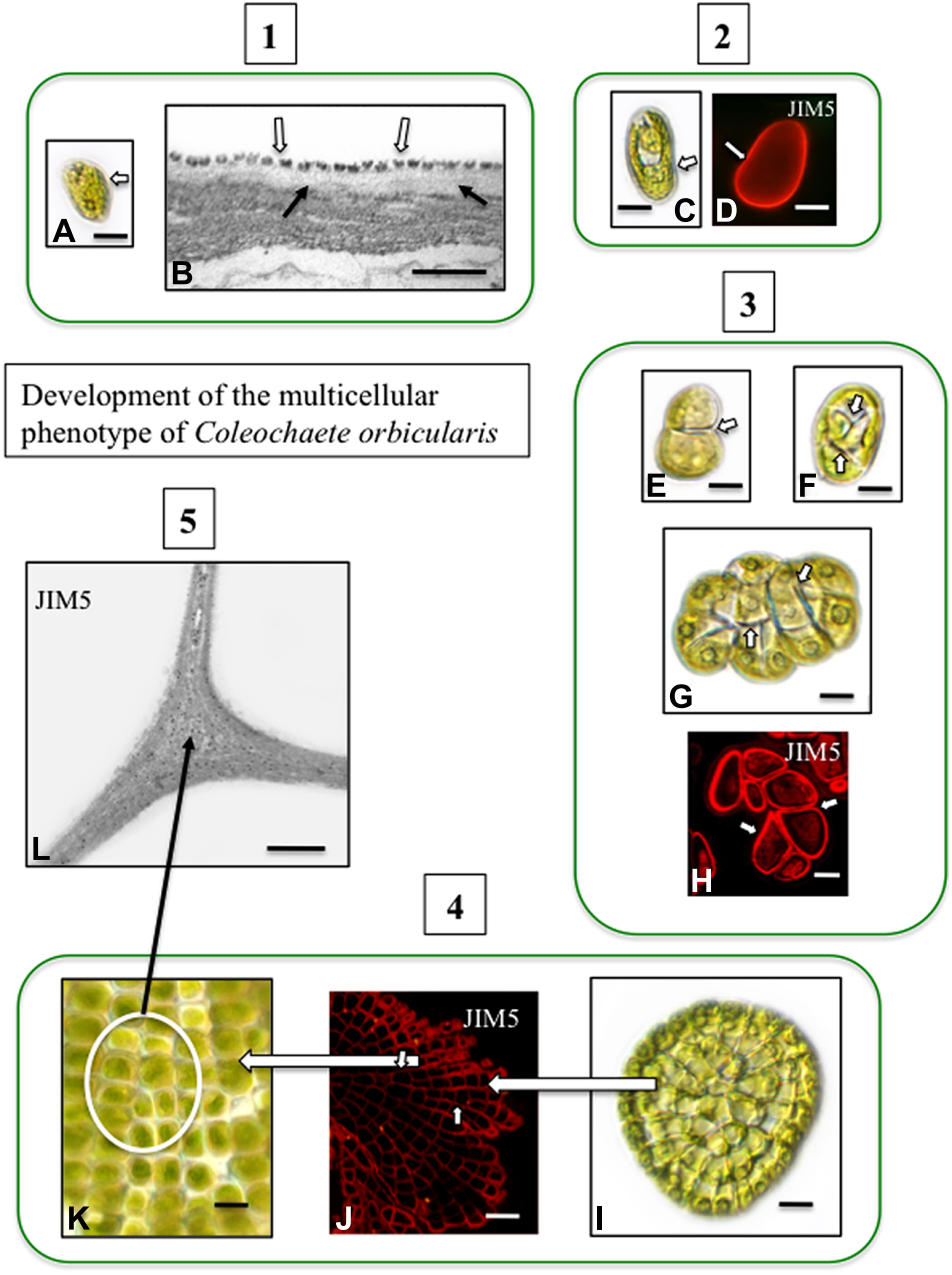
**Development of the multicellular phenotype in *Coleochaete orbicularis*.** (1) Unicellular zoospores **(A)** will swim for several hours before settling down (DICLM; bar = 7 μm). The plasma membrane of zoospores is covered by a layer of square scales (**B**; hollow arrows). In zoospores that have attached surface, fibrillar cell wall material appears under the scale layer [arrows; transmission electron microscopy (TEM) image bar = 500 nm]. (2) After settling down, a cell wall quickly forms on each zoospore (**C**: DICLM; bar = 5 μm). The wall labels with the monoclonal antibody, JIM5, with specificity for relatively low esterified HG [**D**; arrow; confocal laser scanning microscope (CLSM) image; bar = 4 μm]. (3) During intermediate stages of development, the walled cell divides into a 2-cell stage (**E**, arrow; bar = 3 μm), a 4-cell stage (**F**; arrows; bar = 4 μm) and an 8-cell stage (**G**, arrows; bar = 6 μm). Images (**E–G)** are DICLM images. The cell walls of each cell of these multicellular stages (arrows) label with JIM5 (**H**, arrows; CLSM; bar = 3 μm). (4) Late stages of multicellular development. The mature thallus consists of several 100 cells organized in a flattened disc (**I**; DICLM; bar = 10 μm). The cells of this thallus label with JIM5 (**J**; arrows; CLSM; bar = 20 μm). The individual cells of the thallus are closely packed in the disk (**K**; circle inset; DICLM; bar = 7 μm). (5) TEM immunogold labeling with JIM5 highlights HG in the walls of cell junctions (**L**; arrows; bar = 500 nm).

**FIGURE 3:**
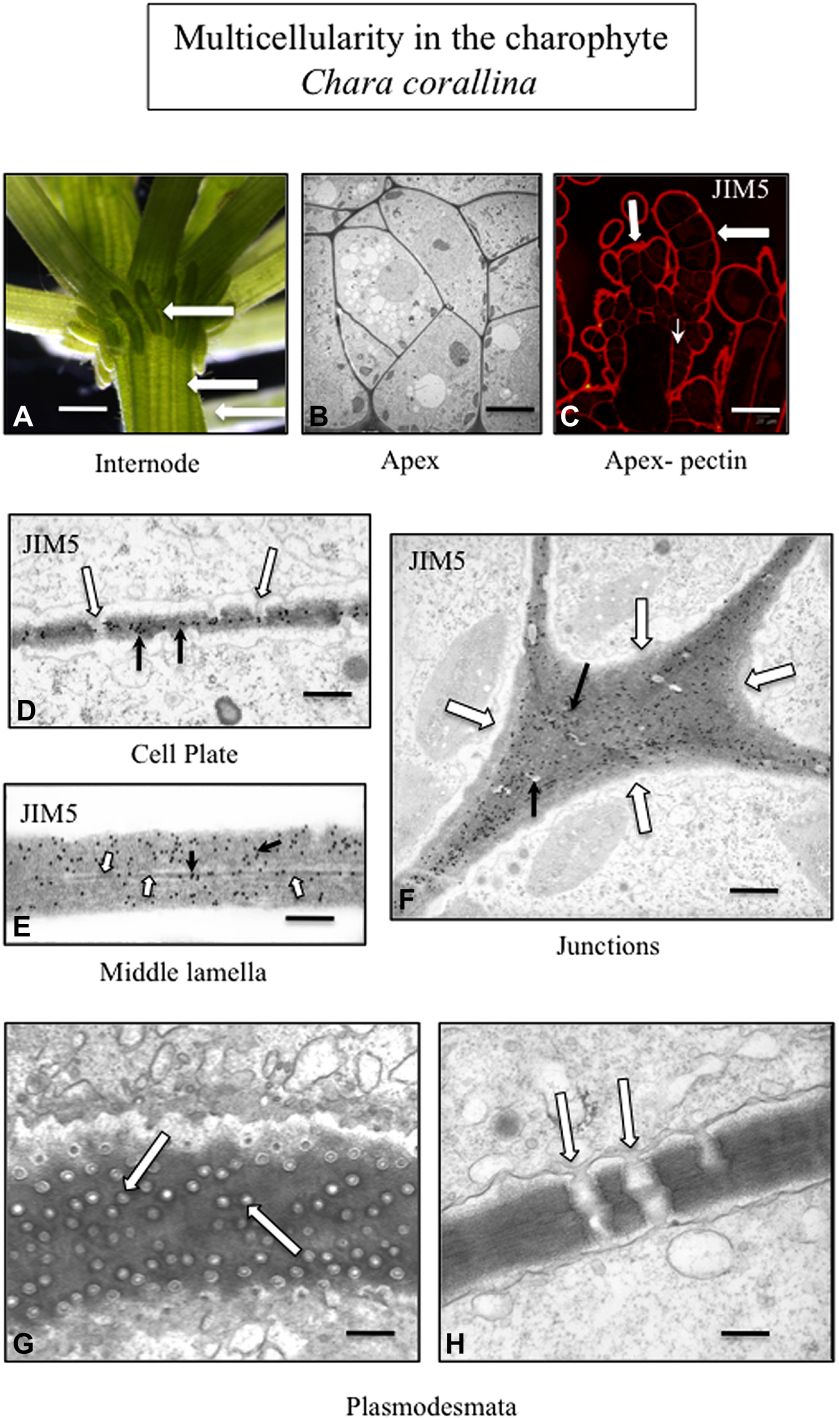
**Multicellularity in the charophyte *Chara corallina.*** The thallus consists of filaments tightly appressed to each other (**A**; arrows; LM; bar = 600 μm). TEM imaging highlights the tight packing of the cells of the thallus (**B**; bar = 4 μm). The cell walls of the outer cells of the thallus (large arrows) and the cross walls (small arrow) label with JIM5 (**C**; CLSM; bar = 200 μm). TEM immunogold labeling with JIM5 (black arrows) of a developing cell plate during cytokinesis. In addition, plasmodesmata (white arrows) are forming during this stage of the cell division (**D**; bar = 100 nm). The wall–wall zone of adjacent cells labels with JIM5 (**E**; white arrows; TEM; bar = 80 nm) and the middle lamella is also apparent (black arrow). The cell walls of the junction zone between cells (**F**; white arrows; TEM; bar = 400 nm) label intensely with JIM5. In the thallus, multiple plasmodesmata penetrate the cell walls and connect adjacent cells (**G, H**; arrows; TEM; **G** bar = 200 nm; **H** bar = 300 nm).

Pectin and pectin modifications including HG and HG cross linking with Ca^2+^, have been well-characterized in charophytes (Proseus and Boyer, 2008; Domozych et al., 2014) and is found in the junction zones between adjacent cells (Domozych et al., 2009a). In *Coleochaete*, zoospores do not have a cell wall but rather a layer of small scales. When zoospores settle down and divide to form multicellular thalli, their scales are sloughed off and are replaced by a pectin-rich cell wall (**Figure 2**). HG is also a major component of the cell walls of *Chara* (**Figure 3**) and is commonly found in zygnematalean taxa including desmids (Domozych et al., 2007, 2009b; Eder and Lütz-Meindl, 2008, 2010). In the desmid, *Penium*, Ca^2+^-complexed HG forms the distinctive outer wall lattice. Its localized secretion and incorporation in the cell wall is a major event associated with the cell’s unique polar expansion mechanism (Domozych et al., 2014). This pectin-based expansion mechanism appears to be very similar to the pectin modifications found in the middle lamella of land plants. This supports the idea that cell–cell adhesion in embryophytes and most likely in multicellular charophytes, evolved by modification of the functional mechanism of cell wall expansion operating in cell wall progenitors (Niklas and Newman, 2013).

The creation of these special zones of cell walls in strategic areas of cell surfaces in order to maximize adhesion efficacy is paramount to multicellular plants. This requires precisely timed and coordinated interactions between the membrane trafficking networks including exocytosis and endocytosis, the cytoskeletal system and particular domains of the plasma membrane/apoplast containing wall modulating enzymes and other effector molecules. Furthermore, all of this must synchronize with highly complex internal regulatory cascades that are part of developmental cycles and be capable of modification in response to environmental stress. Much of our understanding of these events has been based on land plants, particularly angiosperm model organism such as *Arabidopsis*. However, recent studies clearly show that many of these wall morphogenetic mechanisms are found in modern day charophytes and most likely were available to the charophyte that successfully ventured onto land 450–500 million years ago. For example, phragmoplast-based cytokinesis provides the structural framework and target zone for subcellular activities leading to cell plate formation that separates post-mitotic daughter nuclei and creates a new cross wall including a new middle lamella (Segui-Simarro et al., 2004; Jürgens, 2005; Ho et al., 2011). At the termini of this expanding cell plate, recently secreted wall polymers including HG derived from both daughter cells intermingle before becoming consolidated to form an adhesive 3-dimensional network. Phragmoplast/cell plate-based cytokinesis is also found in late divergent charophytes (Pickett-Heaps et al., 1999: Doty et al., 2014) and results in the formation of a new cross wall with a middle lamella. In land plants, modifications in ER distribution during cell plate formation lead to the creation of plasmodesmata, the conduits for intercellular transport and intercellular communication in multicellular plant systems (Lee and Sieburth, 2010; Fitzgibbon et al., 2013). Late divergent charophytes that employ phragmoplast-based cytokinesis (e.g., Charales and Coleochaetales) also produce plasmodesmata (**Figure 3**; Franceschi et al., 1994; Cook and Graham, 1999; Graham et al., 2000). In land plants, when expansion occurs in adjacent cells after cell division, pectin and other wall polymers are directed to specific loci of the cell surface (middle lamella and cell junctions) and must diffuse through the interstices in the outermost microfibril layer of each of the cell walls (Wolf and Greiner, 2012; Bashline et al., 2014). This is necessary to maintain the cell–cell connection. Multicellular charophytes also display focused incorporation of polymers into the expanding cell wall (Proseus and Boyer, 2008). In multicellular plants, the plane of cell division and placement of the new cross wall/middle lamella is predicted by a transient band of cortical microtubules known as the preprophase band (PPB; Vos et al., 2004; Muller et al., 2009; Rasmussen et al., 2010). The PPB functions in cells by controlling the “insertion” of spatially determined new walls within the pattern of neighboring cells: (i.e., a feature of multicellular land plants; Pickett-Heaps et al., 1999). However, recently, a PPB has also been shown to define the future site of cell division and cell expansion in the charophyte, *Penium* (Ochs et al., 2014). In summary, many of the cell wall-based developmental phenomena associated with plants are also found in multicellular charophytes.

## CONCLUDING REMARKS

Multicellularity encompasses complexities that we are only beginning to resolve in green algae and plants. It is very clear that the expression and maintenance of the multicellular form is directly associated with the cell wall. It is also apparent that the composition and microarchitecture of the cell walls of green algae are very diverse but different polymer-based constructions have been utilized to maintain multicellularity. Many specifics in this phenomenon have yet to be discovered but current efforts in the screening of wall polymers and sequencing of genomes offer hope that the “mysteries” will soon be solved.

## Conflict of Interest Statement

The authors declare that the research was conducted in the absence of any commercial or financial relationships that could be construed as a potential conflict of interest.
